# Neither Postabsorptive Resting Nor Postprandial Fat Oxidation Are Related to Peak Fat Oxidation in Men With Chronic Paraplegia

**DOI:** 10.3389/fnut.2021.703652

**Published:** 2021-07-26

**Authors:** Kevin A. Jacobs, David W. McMillan, Jennifer L. Maher, James L. J. Bilzon, Mark S. Nash

**Affiliations:** ^1^Department of Kinesiology and Sport Sciences, University of Miami, Coral Gables, FL, United States; ^2^Christine E. Lynn Rehabilitation Center for the Miami Project to Cure Paralysis, UHealth/Jackson Memorial, Miami, FL, United States; ^3^Department of Physical Medicine and Rehabilitation, University of Miami Leonard M. Miller School of Medicine, Miami, FL, United States; ^4^Department for Health, University of Bath, Bath, United Kingdom; ^5^Departments of Neurological Surgery and Physical Therapy, University of Miami Leonard M. Miller School of Medicine, Miami, FL, United States

**Keywords:** spinal cord injury, fat utilization, fuel metabolism, peak fat oxidation, cardiorespiratory fitness

## Abstract

The peak rate of fat oxidation (PFO) achieved during a graded exercise test is an important indicator of metabolic health. In healthy individuals, there is a significant positive association between PFO and total daily fat oxidation (FO). However, conditions resulting in metabolic dysfunction may cause a disconnect between PFO and non-exercise FO. Ten adult men with chronic thoracic spinal cord injury (SCI) completed a graded arm exercise test. On a separate day following an overnight fast (≥ 10 h), they rested for 60 min before ingesting a liquid mixed meal (600 kcal; 35% fat, 50% carbohydrate, 15% protein). Expired gases were collected and indirect calorimetry data used to determine FO at rest, before and after feeding, and during the graded exercise test. Participants had “good” cardiorespiratory fitness (VO_2peak_: 19.2 ± 5.2 ml/kg/min) based on normative reference values for SCI. There was a strong positive correlation between PFO (0.30 ± 0.08 g/min) and VO_2peak_ (*r* = 0.86, *p* = 0.002). Additionally, postabsorptive FO at rest was significantly and positively correlated with postprandial peak FO (*r* = 0.77, *p* = 0.01). However, PFO was not significantly associated with postabsorptive FO at rest (0.08 ± 0.02 g/min; *p* = 0.97), postprandial peak FO (0.10 ± 0.03 g/min; *p* = 0.43), or incremental area under the curve postprandial FO (*p* = 0.22). It may be advantageous to assess both postabsorptive FO at rest and PFO in those with SCI to gain a more complete picture of their metabolic flexibility and long-term metabolic health.

## Introduction

Spinal cord injury (SCI) incites persistent metabolic derangement, readily observed through the interaction of decreases in the mass of skeletal muscle (1) and bone (2) and increases in fat mass (3). These profound changes in body composition coalesce into an obese phenotype of neurogenic origins. Thus, the Consortium for Spinal Cord Medicine Clinical Practice Guidelines for Cardiometabolic Disease in persons with SCI identify obesity as the most prevalent component risk in this population (4) and find that other dyslipidemic risk factors cluster in a manner favoring disordered fat metabolism (5). While the etiology of obesity with SCI is multifactorial, impaired metabolic flexibility, the reduced capacity to respond to a metabolic challenge by transitioning between carbohydrate and fat utilization, likely plays a critical role. In the general population, metabolic flexibility appears to be a good indicator of long-term metabolic health. Individuals with a high 24-h respiratory quotient (RQ) measured when they were in energy balance (indicative of a lower reliance on fat), were more likely than those with a low 24-h RQ to gain significant amounts of body weight and fat mass over a 2 to 6-year period (6–9). Others, however, have not observed this relationship (10). More recently, it was shown that poor metabolic flexibility in the face of 24-h high-fat overfeeding predicted future weight gain (11). While these studies are intriguing, the 24-h measurements rely on whole-room calorimeters, limiting the implementation of this resource-intensive method by practitioners to identify those most at risk for poor metabolic health outcomes.

Metabolic flexibility can be examined not only during an acute dietary challenge, but also during acute exercise. The peak rate of fat oxidation (PFO) and the intensity at which it occurs can be identified using a graded exercise test to exhaustion and indirect calorimetry (12). The effects of diet, training status, and sex on PFO have been well-studied (13). More recent work has shown that postabsorptive fat oxidation (FO) at rest (14, 15), 24-h FO, and insulin sensitivity (16) in healthy men are positively related to PFO. These relationships have important health implications as they provide evidence identifying PFO as an accessible and affordable means of assessing metabolic flexibility and long-term metabolic health.

Persons with SCI have a low PFO that occurs at a low relative exercise intensity (17–20). These findings are likely the result of physiological effects of SCI that reduce mobilization, delivery, and uptake of fat during exercise (21). Additionally, the upper body mode of voluntary exercise those with SCI are limited to is less reliant on fat than leg exercise (22, 23). Despite the well-established correlation between PFO and non-exercise FO in the general population, in persons with SCI the relationship between FO in the postabsorptive rest and postprandial states, the primary constituents of 24-h FO, and PFO are unknown.

The purpose of this study was to determine the relationship between FO in the postabsorptive rest and postprandial states and PFO in those with SCI. The degree to which these variables are related may determine whether PFO can be used to predict the future metabolic health of those with SCI and to target effective interventions to those most in need.

## Methods

This manuscript presents unpublished data from a larger study examining the acute effects of exercise mode and intensity on postprandial metabolism with a repeated measures design in those with chronic paraplegia (24). As such, details of the Methods have been previously published (24, 25) and relevant details are presented here.

### Participants

We recruited 11 participants based on an a-priori sample size calculation (Cohen's *d* = 1.0, α = 0.05, ß = 0.90) to detect a significant difference in the change of insulin AUC between different modes and intensities of exercise (25). Following baseline assessments, one participant was withdrawn from the study after being prescribed a medication for type 2 diabetes by his physician. Thus, 10 adult men with chronic thoracic spinal cord injury (SCI) participated in the study (Table 1). Participants were included if they were male, aged ≥ 18 years old with neurologically stable spinal cord injury (ASIA Impairment Scale A-C) at T1 and lower spinal levels for > 1 year, were not taking any medications and did not have any illness (e.g., diabetes) that would interfere with metabolic outcome variables, and were able and willing to comply with study procedures. None of the subjects were insulin resistant as estimated using the HOMA-2 model (24). The procedures and risks were thoroughly explained to the participants and their written, voluntary, informed consent was obtained. The study is registered with ClinicalTrials.gov (NCT03545867) and procedures were published (25) and are in accordance with the Human Subjects Research Office, University of Miami Miller School of Medicine.

**Table 1:** Participant characteristics.

**Participant**	**Age (yr)**	**Stature (m)**	**Body Mass (kg)**	**Injury Duration (yr)**	**Injury Level**	**AIS**
1	28	1.68	72.6	10	T2	A
2	45	1.73	78.4	16	T6	A
3	37	1.88	99.5	19	T4	A
4	28	1.70	51.2	8	T6	A
5	51	1.65	65.6	8	T10	A
6	32	1.83	67.6	15	T3	A
7	35	1.78	80.8	3	T4	B
8	38	1.74	106.5	13	T6	C
9	57	1.70	64.9	34	T8	B
10	38	1.73	62.5	6	T9	A
Average	39 ± 10	1.74 ± 0.07	75.0 ± 17.0	13.2 ± 8.8		

*Values are mean ± SD; n = 10. yr, year; m, meters; kg, kilograms; AIS, ASIA (American Spinal Injury Association) Impairment Scale, where A is complete with no motor or sensory function preserved below the level of the injury, B is incomplete with sensory function preserved but no motor function preserved below the level of the injury, C is incomplete with motor function preserved below the level of the injury and more than half of key muscles below the level of injury have a muscle grade of 3 or more (26)*.

### Aerobic Capacity and Peak Fat Oxidation During Exercise

Participants were instructed to refrain from caffeine, alcohol ingestion, and strenuous exercise for 24 h before testing and to arrive at the laboratory normally hydrated (500 ml of water within 1 h of testing) following an overnight fast (≥10 h). Cardiorespiratory fitness (VO_2peak_) and substrate oxidation rates were assessed using a continuous, graded, voluntary, arm cycle exercise (Lode Angio, Groningen, Netherlands) test (GXT) to volitional exhaustion. Participants began cycling at 0–50 W (mean ± SD: 28 ± 8 W) depending on exercise history to elicit volitional exhaustion in 8–12 min. Exercise was performed at a constant cadence of 60 ± 5 rpm and the workload was increased by 20 W every 3 min until the participants asked to stop or could not maintain a cadence above 45 rpm. Similarly trained participants with SCI reached metabolic steady state within 3 min during graded arm cycling exercise (27). Expired gases were continuously collected throughout exercise using a fitted Hans-Rudolph oro-nasal mask and analyzed by a portable open-circuit indirect calorimetry system (Oxycon Mobile, Viasys, Inc.). Indirect calorimetry data and stochiometric equations (28) were used to determine FO during exercise. The PFO was defined as the highest rate of FO achieved in 20-s averages of data collected during the graded exercise test.

### Substrate Use During Postabsorptive Rest and Postprandial Conditions

Participants recorded their diet for 24 h before the measurement of substrate oxidation during postabsorptive rest and postprandial conditions, and during this time were asked to refrain from caffeine, alcohol ingestion, and strenuous exercise. On the morning of the trial, participants were instructed to consume ~10 ml/kg of water on waking and report to the laboratory following an overnight fast (≥10 h). Upon arrival, participants were fitted with a mask (as described above) and remained seated in their wheelchair for 60 min while expired gases were collected at two different time points. Participants then consumed a liquid mixed meal (600 kcal; 35% fat, 50% carbohydrate, 15% protein) in ≤ 6 min. The meal consisted of one banana, 42.5 g of glucose polymer powder, 32 g of Jif creamy peanut butter, 7 g of coconut oil, and 19.5 g of whey protein isolate powder. The macronutrient distribution of this meal was designed to be equal to *ad libitum* published norms in SCI (29). Expired gases were collected every 30 min during the 150-min postprandial period while participants remained seated in their wheelchair. As described, indirect calorimetry data collected during the postabsorptive rest and postprandial periods were analyzed by a portable open-circuit indirect calorimetry system and rates of energy expenditure (EE) as well as CHO oxidation and FO were calculated using stochiometric equations (28).

### Statistical Analysis

For postprandial time series data, incremental area under the curve was calculated using denominations of the trapezoidal rule using an open-source tool deployed via Microsoft Excel (version 2103) (30). Fat oxidation data from the GXT and postabsorptive/postprandial EE data were analyzed by repeated measures one-way analysis of variance (time) followed by *post hoc* analyses using the least significant difference test when appropriate. Fat oxidation data from stages 4 and 5 of the GXT were excluded from statistical analyses because of the low number of subjects that completed those stages (see Results). Relationships between variables were quantified by Pearson correlation. Statistical significance was set at an α level of *p* < 0.05 and data are presented as mean ± SD. Data organization, analysis, and visualization were performed via Microsoft Excel (version 2103), with plots imported into Adobe Illustrator (version 25.0) to create the final figures.

## Results

During the GXT, participants reached an average peak power output of 108 ± 25 W (81–164 W), HR_peak_ of 169 ± 16 beats/min (134–188 beats/min), and VO_2peak_ of 19.2 ± 5.2 ml/kg/min (12.8–31.8 ml/kg/min). The participants had “good” cardiorespiratory fitness based on normative data for paraplegic men (31).

**Figure 1:**
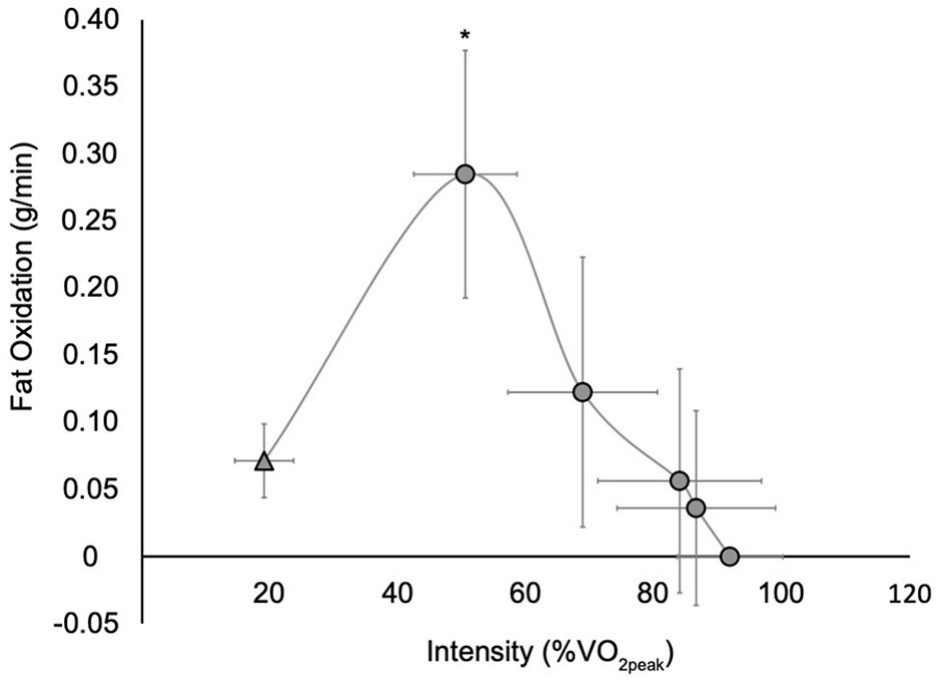
Whole body fat oxidation at rest (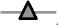) and during a graded exercise test (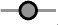). Values are mean ± SD; *n* = 10. VO_2peak_, peak oxygen consumption. All participants completed rest and stages 1 through 3, while four participants completed stage 4 and two participants completed stage 5. *Significantly different than rest and stages 2–3 (*p* < 0.001) (stages 4 and 5 were not included in the analysis as few participants completed these stages).

The rate of FO increased more than 3-fold from rest to an average PFO during the GXT of 0.30 ± 0.08 g/min (0.22–0.46 g/min) that occurred at 50.7 ± 8.1% VO_2peak_ (38.9–66.6% VO_2peak_) (Figure 1). There was a main effect of time (F_3,27_ = 33.1, p < 0.001) with *post hoc* analyses revealing that FO values during stage 1 of the GXT were significantly greater than those at rest and stages 2–3 (*p* < 0.001). All 10 participants reached PFO during the first stage of the GXT and could complete stages 1–3. Three of the four participants that completed stage 4 reached a FO rate of zero during this stage. The two participants that completed stage 5 both reached a FO rate of zero during this stage.

**Figure 2:**
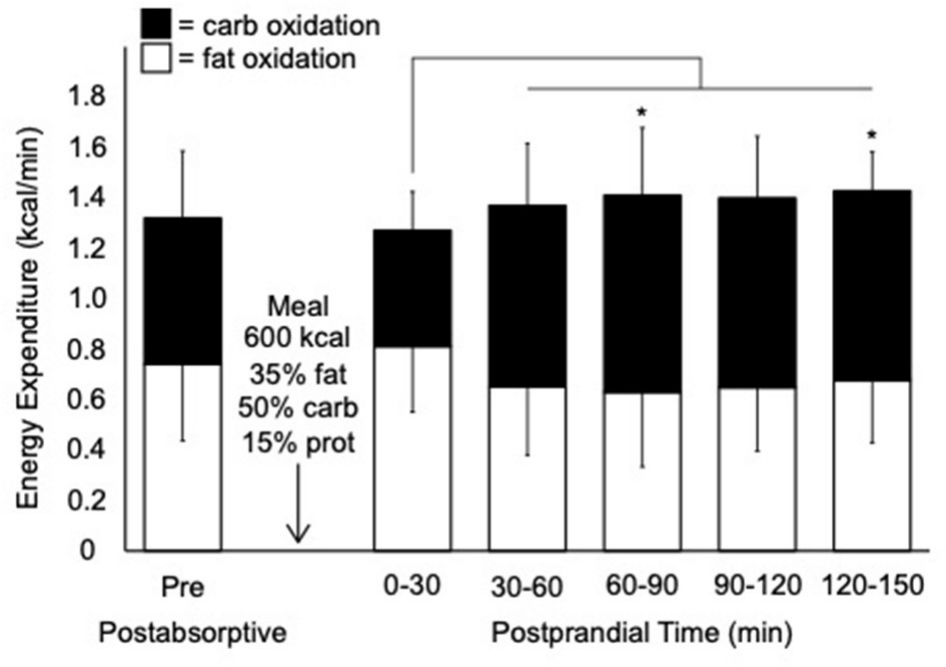
Rates of total, carbohydrate, and fat expenditure before and after consumption of a liquid mixed meal. Values are mean ± SD; *n* = 10. kcal; kilocalorie; carb, carbohydrate; prot, protein. Thin line denotes a significant increase in carbohydrate oxidation compared to 0–30 min postprandial (*p* < 0.05). *Significant difference in carbohydrate oxidation compared to postabsorptive pre (*p* < 0.05).

In the 24 h before the postprandial test, participants consumed 1,942 ± 15 kcal/day (39% CHO, 37% fat, and 22% protein). The following morning in the postabsorptive resting state, the contributions of fat and CHO to total EE were 56 and 44%, respectively (Figure 2). The average postabsorptive FO rate at rest was 0.08 ± 0.02 g/min (0.03–0.11 g/min).

During the postprandial period, there was a trend for a main effect of time for total EE (F_5,45_ = 4.0, *p* = 0.076) (Figure 2). There was a main effect of time for carbohydrate oxidation (F_5,45_ = 15.2, *p* = 0.004) with a significant increase in carbohydrate oxidation after the first (0–30 min) postprandial time point. There was a trend for a main effect of time for FO (F_5,45_ = 4.6, *p* = 0.062). The average peak FO during the postprandial period was 0.10 ± 0.03 g/min (0.07–0.16 g/min).

**Figure 3:**
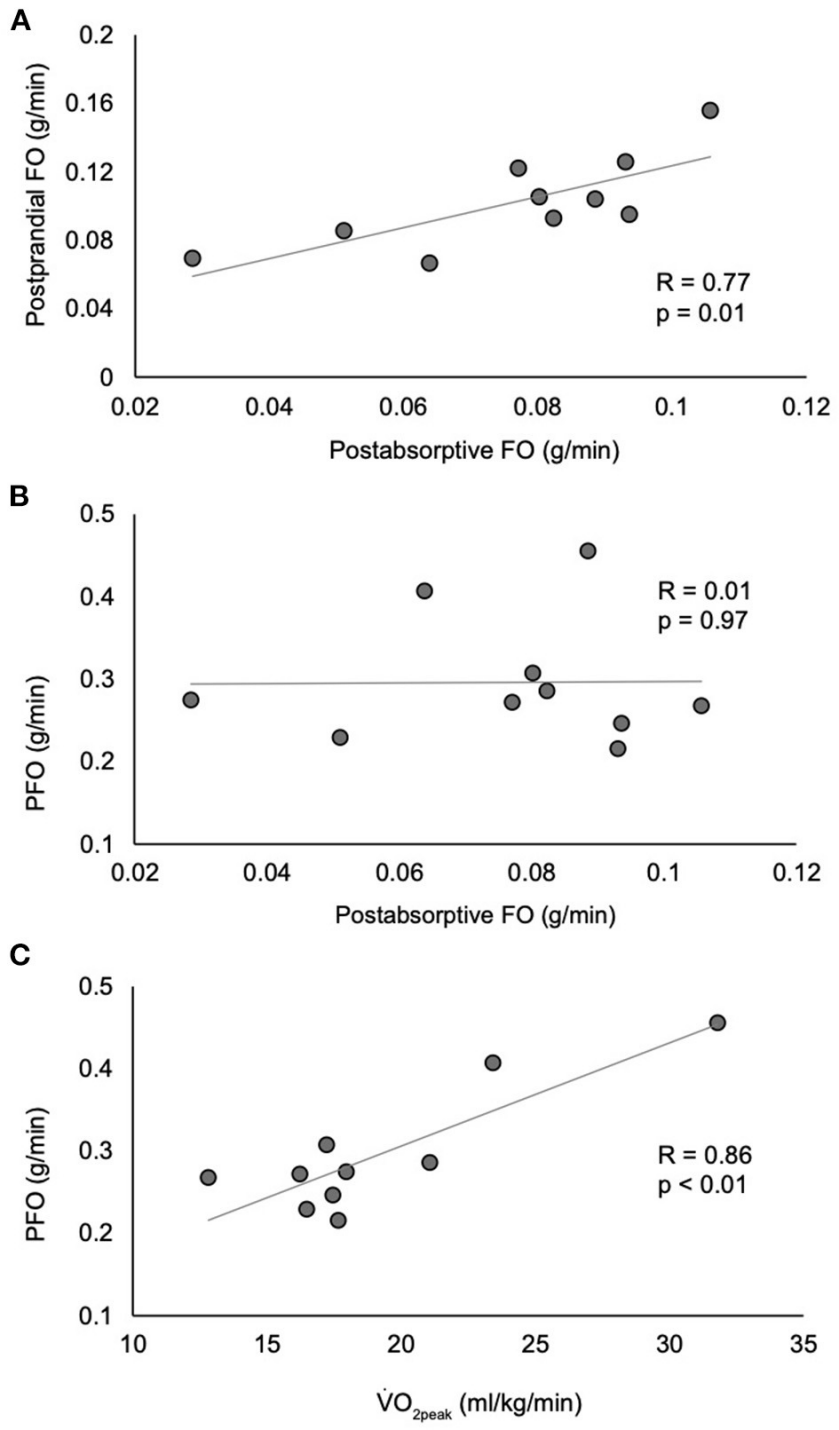
Correlations between postabsorptive fat oxidation and postprandial fat oxidation **(A)**, peak fat oxidation **(B)**, and between VO_2peak_ and peak fat oxidation **(C)**. *n* = 10. FO, fat oxidation; PFO, peak fat oxidation; VO_2peak_, peak oxygen consumption.

While postabsorptive FO at rest was significantly and positively correlated with postprandial peak FO (*r* = 0.77, *p* = 0.01), there was no correlation between postabsorptive FO at rest and PFO (*r* = 0.01, *p* = 0.97) (Figure 3). Additionally, there was no correlation between postprandial peak FO and PFO (*r* = −0.28, *p* = 0.43) or incremental area under the curve postprandial FO (*p* = 0.22). Neither age (*r* = 0.14, *p* = 0.71) nor BMI (17.7–35.2 kg/m^2^, *r* = −0.29, *p* = 0.42) were correlated with PFO. However, there was a strong positive correlation between VO_2peak_ and PFO (*r* = 0.86, *p* = 0.002).

## Discussion

The purpose of this study was to determine the relationship between FO in the postabsorptive resting and postprandial states and PFO in males with chronic SCI. The primary finding was that neither postabsorptive FO at rest nor postprandial peak FO were related to PFO. Instead, significant and positive correlations were found between FO in the postabsorptive resting and postprandial states and between VO_2peak_ and PFO. These findings highlight the unique influences of upper body exercise and/or the metabolic effects of SCI that may result in a dissociation between FO in the postabsorptive resting and postprandial states and PFO.

There is large interindividual variation in FO in the postabsorptive resting state with trained men exhibiting RER values as wide-ranging as 0.72–0.93 (32). Similarly, the moderately fit paraplegic men enrolled in the current study had RER values ranging from 0.77 to 0.92 in the postabsorptive resting state. The primary determinants of FO in the postabsorptive resting state are fuel availability (muscle glycogen content and plasma concentrations of FFA and lactate), skeletal muscle morphology (proportion of type 1 fibers), and behavioral factors (training volume and percent of dietary fat intake) (32). Hereditary factors play a larger role in determining FO at rest than physical activity level (33) and body composition appears to have little influence (32). Despite the known reductions in the proportion of type 1 muscle fibers (34–38) and capacity to increase exercise energy expenditure (39) with SCI, the rates of FO of our participants in the postabsorptive resting (0.08 ± 0.02 g/min) and postprandial states (0.10 ± 0.03 g/min) are very similar to those of non-injured participants (0.06–0.08 and 0.08–0.12 g/min, respectively) (14, 33, 40, 41). The participants of the current study were able to achieve normal rates of FO in the face of the moderate metabolic challenges of fasting and consumption of an acute mixed meal. However, SCI presents a unique set of challenges when faced with the greater metabolic challenge of exercise.

Fat oxidation during exercise in those with SCI is limited by the mode of voluntary exercise adopted and the unique effects of SCI on metabolism. Persons with SCI performing voluntary exercise are often limited to use of the upper body that, with its 2- to 3-fold smaller muscle mass than the legs (42), is less capable of achieving high rates of FO. Additionally, the arms are less efficient than the legs in extracting oxygen due to greater variability in blood flow and diffusion limitations (22, 42) and less capable of oxidizing fat (22, 23) due to lower beta oxidative enzyme activities (43). The decreased ability to oxidize fat during upper body exercise is compounded by SCI as it results in the reduced mobilization, delivery, and uptake of fat during exercise compared to those without SCI (21). While we did not include non-injured participants in this study, we previously showed that sedentary men with paraplegia had higher PFO values than healthy non-injured participants when both groups performed an arm ergometer GXT (18). These results indicate that the low rates of FO during voluntary arm exercise in those with paraplegia may be more a function of the mode of exercise than SCI *per se*.

The PFO values of the participants in the current study (0.30 ± 0.08 g/min) are similar to those of endurance-trained men with SCI (0.22–0.28 g/min) (19, 20) and significantly higher than those of sedentary paraplegic men (0.13 ± 0.07 g/min) (18). Despite the limitations to FO in those with SCI performing upper body exercise, the PFO values of this study compare well with non-injured, untrained, normal or overweight men and women performing leg exercise, with PFO values of 0.17–0.33 g/min (15, 33, 44, 45). The PFO values of non-injured healthy and recreationally-to- moderately trained men (0.46–0.60 g/min) are higher than those reported here (12, 14, 16, 46, 47).

The close relationship between PFO and 24-h FO (16) and FO in the postabsorptive state at rest in non-injured individuals (14, 15) has led to the belief that PFO has the potential to be an accessible and affordable means of assessing metabolic flexibility and long-term metabolic health. However, in the current study, there is no relationship between FO in the postabsorptive resting state and PFO (Figure 3B), drawing into question the usefulness of PFO as a marker of metabolic flexibility in those with SCI. The discordance in the results of our study and those of non-injured individuals is likely due to large methodological differences. The studies reporting a close relationship between FO in the postabsorptive resting state and PFO involved 57 recreationally active, non-injured men performing treadmill exercise while our study involved 10 paraplegic men with good fitness performing arm ergometry. While the range of FO values in the postabsorptive resting state were similar between our study (0.03–0.11 g/min) and the non-injured studies (0.02–0.15 g/min) (14, 16), the range of PFO values varied greatly (0.22–0.46 vs. 0.30–1.02 g/min, respectively). Thus, our comparison of fewer data points within a relatively narrow range of PFO values may explain a lack of a relationship between FO in the postabsorptive resting state and PFO. Future work that includes non-injured controls will be very helpful in interpreting the individual effects of exercise mode and SCI on PFO and its relationship to FO in the postabsorptive resting state.

The wide ranges of age, BMI, injury duration, and VO_2peak_ of the participants in this study may have contributed to increased variability in the data collected. However, the goal of this study was to generalize the results to those with chronic paraplegia, a population that is inherently heterogenous. Our study was limited by not including any female participants. Non-injured men have a higher absolute PFO than women, while PFO expressed relative to fat free mass is higher in women than men (46, 47). Sex differences in PFO among those with SCI are unknown and need to be examined in future studies. Additionally, it would have been optimal to feed our participants a meal with an energy content that was standardized relative to fat-free mass. Without any body composition data, we felt that it was best to provide a standard meal with an energy content that was similar to what our participants consumed habitually. Finally, our finding that PFO occurred in the first stage of the GXT may mean that in some participants we missed the workload that elicited PFO and should have used a more gradual increase in workload at the beginning of the test.

In conclusion, assessing the metabolic flexibility of those with SCI is complicated by their reliance on upper body exercise and the unique metabolic effects of their injury. In paraplegic men with good cardiorespiratory fitness, there appears to be a dissociation between postabsorptive FO at rest and PFO achieved during a graded exercise test. These results suggest that it may be advantageous to assess both postabsorptive FO at rest and PFO in those with SCI to gain a more complete picture of their metabolic flexibility and long-term metabolic health.

## Data Availability Statement

The raw data supporting the conclusions of this article will be made available by the authors, without undue reservation.

## Ethics Statement

The studies involving human participants were reviewed and approved by Human Subjects Research Office, University of Miami Miller School of Medicine. The patients/participants provided their written informed consent to participate in this study.

## Author Contributions

KJ contributed to study design, data analysis and interpretation, and original draft preparation of the manuscript. DM, JM, and JB contributed to study design, data collection, data analysis and interpretation, and manuscript review and editing. MN contributed to study design, data analysis and interpretation, and manuscript review and editing. All authors contributed to the article and approved the submitted version.

## Conflict of Interest

The authors declare that the research was conducted in the absence of any commercial or financial relationships that could be construed as a potential conflict of interest.

## Publisher's Note

All claims expressed in this article are solely those of the authors and do not necessarily represent those of their affiliated organizations, or those of the publisher, the editors and the reviewers. Any product that may be evaluated in this article, or claim that may be made by its manufacturer, is not guaranteed or endorsed by the publisher.
